# Sir George Newman's Annual Report

**Published:** 1921-07-23

**Authors:** 


### THE PUBLIC WELL-BEING.

### Sir George Newman's Annual Report.

THE annual report of Sir George Newman, the Chief Medical Officer of the Ministry of Health, for the year 1920 has now been published by the Stationery Office (price 1s. 6d.). It is entitled "On the State of the Public Health," and deals comprehensively and in a popular manner with the state of public health during 1920 as revealed by official statistics and by the work and records of the Ministry. With regard to the future, Sir George Newman is insistent upon two points, that the watchword of our medical services should be for the moment the "intensive culture" of fields already tilled rather than the exploitation of fresh pastures, and that every pound expended on our medical services must be made to yield the full value of a pound.

During 1920 the report shows there was a substantial rise in the birth-rate, which reached the figure of 25.4, as compared with 18.5 in 1919. The death-rate (12.4) shows a steady decline at most ages. The infant mortality rate (80 per 1,000 births) is the lowest ever recorded, though there is still much unnecessary loss of life, both of mother and infant. It is noted that cases of epidemic and infectious disease exceed half a million per annum, and though the primary burden is relatively light, yet the ultimate results are grave. There is also a large mass of disability and incompetence from relatively trivial sickness, and the expenditure on sickness and disablement benefit denotes an amount of time lost from employment amounting to no less than the equivalent of one year's labour of 270,000 persons for the insured population alone. This is a grave position, and should give pause to those who advocate reduced expenditure on measures which concern the physical welfare of the young.

### MATERNITY AND CHILD-WELFARE.

The fall recorded in infant mortality is held to be due not so much to any one factor as to general enlightenment and the co-ordination of ameliorative agencies on behalf of the mother. As a direct illustration figures are quoted showing that with the increase of the number of infant welfare centres from 340 in 1914 to 1,937 in 1920, the infant mortality rate has fallen from 105 to 80 per thousand during the same years. About 50,000 mothers bring their babies to the centres during a week; the number of mothers going regularly to centres is probably not less than 150,000. At least one-sixth of the total babies born come directly under the supervision of doctor and nurse, in addition to a much larger number visited at their own homes. Maternal deaths shows an increase during 1919 and 1920, and the need for more maternity accommodation is insisted upon.

### INSURANCE AND MEDICAL SERVICE.

During 1920 revised conditions of service for insurance practitioners were put into operation. A new scale of medical remuneration was introduced, the new medical record cards were brought into use, and a regional medical staff of thirty officers was appointed. These reforms, mostly suggested and approved for some years, had been delayed by the war and aim at an improvement in the efficiency of the treatment of the insured sick, who numbered in 1920, in England and Wales, 13,873,000.

### EPIDEMIC AND INFECTIOUS DISEASES.

The common epidemic diseases of the country, though not now usually a chief cause of death, are a serious cause of sickness and one of the principal causes of invalidism. During 1920 only 274 cases of small-pox were notified, all of a mild type, and no deaths. Scarlet fever prevailed more extensively than in any year since 1915 (119,490 cases and 1,430 deaths). Diphtheria was more prevalent than for some years (69,481 notifications and 5,641 deaths). In general, however, the epidemic prevalence has been characterised by a low case mortality. The report recalls the rise in the number of deaths from influenza during 1920. The difference between the figures for pulmonary tuberculosis in 1920 compared with 1917 shows practically a reduction of over one-sixth, and the total number of cases notified is the lowest recorded since compulsory notification came into force. A substantial fall has also taken place in the number of deaths registered. Though the returns since 1847 are full of encouragement, and witness a steady conquest of the disease, this should not lead to any slackening in the fight against one of the formidable enemies of the race. The Report indicates the means by which it can best be resisted, and outlines the functions of (1) the medical service, (2) the dispensary, (3) residential institutions, (4) after-care, and (5) research work done.

In regard to venereal disease, the policy and practice of the Ministry of Health are based largely on the findings of the Royal Commission of 1916. Except for providing for the confidential registration of the cause of death (found up to the present to be impracticable) the Ministry have carried out all the important recommendations of the Commission and extended their application. The Report contains a summary of the facilities provided for investigation, diagnosis, and treatment, and of the work done. The rapid rise of "new" cases in 1918 and 1919 did not continue in 1920, but there was a considerable increase in the total attendances, and the clinics are more appreciated as time passes.

Sir George Newman ends his report with a call to local authorities. "If authorities," he says, "would only put into wise operation the health legislation they possess before seeking further powers from Parliament, an immense advance, truly conservative and truly constructive, would be made." It is in the hands of the local authority that the main business of the execution of national health policy rests. We commend this Report to the careful study of every thinking man and woman.

